# Nil Admirari

**Published:** 1867-12

**Authors:** 


***Nil Admirari.***

Because the Editorial modesty of the JOURNAL feared to express the exultant feelings of the editorial heart on the occasion of the inauguratory exercises on the completion of the new building for Rush Medical College; the editor refrained from writing an original account thereof, but was content to reproduce the reports to the City press of Chicago, made by wholly impartial witnesses. When one quotes from Scripture or Shakspeare, it is unnecessary to specify the source, for that would be considered an insult to the understanding and intelligence of reader or hearer. A similar state of things prevails with regard to the public press of Chicago. Who does not read it? To whom is it necessary to say this is from the Chicago press?

Years ago we supposed astonishment on our part at anything was a thing of the past. But it appears the Millenium has not yet come. "The people which sit in darkness" do not all of them, as yet, "see great light"—particularly in Cincinnati. They do not read the Chicago newspapers. Hence it happens when the JOURNAL quotes the words of impartial and disinterested reporters for the Chicago secular press—the LANCET, not even usually incisive, is bewildered at the "glorification," supposing it to be hyperbolical or worse. Dear LANCET, shake off your suburban notions for the nonce—come up to Chicago, and out of the fulness of the editorial heart we promise that you shall return to your smoky hamlet on the Ohio, if perchance you should thereafter wish to return, exclaiming, like the Queen of Sheba: The half has not been told!

Speaking of the LANCET—its "managing editor" aims his weapon at the jugulars of Drs. G. and A. with a bold and direct thrust. The reference is to the numbers of different classes in *Western* Medical Colleges. Whilst the LANCET "pauses,"

we reply: Nashville and Louisville (*quantum mutati ab illis!*) are *Southern* schools. Kentucky and Tennessee, in Yankee parlance, have always been "reckoned" Southern States. The classes in those schools, we might add, were ephemeral. Rush College claims a steady increase for a quarter of a century, with scarcely a ripple upon the surface of its fortunes from the rocks and curses hurled by the Apostle of Reform and his confreres.

Reference is made to Ann Arbor, and it is insisted that that concern is a "legitimate" Medical School, "for certes Dr. A. and the big Gunn of Rush were recently strong pillars in that temple."

Soberly and seriously the JOURNAL insists that the Ann Arbor concern is by no means *legitimately* to be compared to Rush or any other Western College, simply because it owes its numbers, in great part, to our knowledge, not to the character of its Faculty, whatever that may be, or to any positive superiority it may afford for medical teaching or illustration, but simply because it charges but a nominal fee for its tickets. The competition being thus wholly and absurdly illegitimate; it ought not to be ranked among legitimately competing schools.

That Dr. A. or Dr. G. made it a gymnasium of preparation for the higher duties involved in their present connection with Rush College, is only another evidence of the truth of the statement of the Chicago press which we quoted, and to which our contemporary refers.

Students at that preparatory department for Medical Colleges, in a large proportion, manifest their appreciation of the same fact by resorting to Rush Medical College, or Cincinnati, to secure the honors of the doctorate.

Meanwhile, Cincinnati, we extend to you the cordial invitation: *Come and see.*

